# Immune features of the peritumoral stroma in pancreatic ductal adenocarcinoma

**DOI:** 10.3389/fimmu.2022.947407

**Published:** 2022-09-05

**Authors:** Azaz Ahmed, Rosa Klotz, Sophia Köhler, Nathalia Giese, Thilo Hackert, Christoph Springfeld, Dirk Jäger, Niels Halama

**Affiliations:** ^1^ Translational Immunotherapy (D240), German Cancer Research Center (DKFZ), Heidelberg, Germany; ^2^ Medical Oncology and Internal Medicine VI, National Center for Tumor Diseases (NCT), University Hospital Heidelberg, Heidelberg, Germany; ^3^ BioQuant, Faculty of Biosciences, Heidelberg University, Heidelberg, Germany; ^4^ General, Visceral and Transplantation Surgery, University Hospital Heidelberg, Heidelberg, Germany; ^5^ Applied Tumor Immunity Clinical Cooperation Unit (D120), National Center for Tumor Diseases (NCT), German Cancer Research Center (DKFZ), Heidelberg, Germany; ^6^ Helmholtz Institute for Translational Oncology Mainz (HI-TRON), Mainz, Germany

**Keywords:** pancreatic cancer, stroma, stromal immunology, IL9, IL18

## Abstract

**Background:**

The peritumoral stroma is a hallmark of pancreatic ductal adenocarcinoma (PDA) with implications for disease development, progression and therapy resistance. We systematically investigated immune features of the stroma in PDA patients to identify markers of clinical importance and potential therapeutic targets.

**Methods:**

Tissue and blood samples of 51 PDA patients with clinical and follow-up information were included. Laser Capture Microdissection allowed us to analyze the stromal compartment in particular. Systematic immunohistochemistry, followed by software-based image analysis were conducted. Also, multiplex cytokine analyses (including 50 immune-related molecules) were performed. Functional analyses were performed using patient-derived 3D bioprints. Clinical information was used for survival analyses. Intercompartmental IL9 and IL18 gradients were assessed in matched samples of tumor epithelium, stroma, and serum of patients. Serum levels were compared to an age-matched healthy control group.

**Results:**

Stromal IL9 and IL18 are significantly associated with patient survival. While IL9 is a prognostic favorable marker (p=0.041), IL18 associates with poor patient outcomes (p=0.030). IL9 correlates with an anti-tumoral cytokine network which connects regulation of T helper (Th) 9, Th1 and Th17 cells (all: p<0.05 and r>0.5). IL18 correlates with a Th1-type cytokine phenotype and stromal CXCL12 expression (all: p<0.05 and r>0.5). Further, IL18 associates with a higher level of exhausted T cells. Inhibition of IL18 results in diminished Th1- and Th2-type cytokines. Patients with high stromal IL9 expression have a tumor-to-stroma IL9 gradient directed towards the stroma (p=0.019). Low IL18 expression associates with a tumor-to-stroma IL18 gradient away from the stroma (p=0.007). PDA patients showed higher serum levels of IL9 than healthy controls while serum IL18 levels were significantly lower than in healthy individuals. The stromal immune cell composition is distinct from the tumor epithelium. Stromal density of FoxP3^+^ regulatory T cells showed a tendency towards improved patient survival (p=0.071).

**Conclusion:**

An unexpected high expression of the cytokines IL9 and IL18 at different ends is of significance in the stroma of PDA and relates to opposing patient outcomes. Sub-compartmental cytokine analyses highlight the importance of a differentiated gradient assessment. The findings suggest stromal IL9 and/or IL18 as markers for patient stratification and as potential therapeutic targets. Future steps include investigating e. g. the role of local microbiota as both cytokines are also regulated by microbial compositions.

## Introduction

Pancreatic ductal adenocarcinoma (PDA) is usually diagnosed at an advanced stage, and it is characterized by an aggressive behavior making it one of the most lethal tumor diseases (1). It is expected that PDA will surpass other entities and become the second leading cause for cancer-related death by 2030 (2). Treatment approaches like chemo- or radiotherapy show little improvement of patient survival and studies investigating immunotherapeutic strategies remained mostly ineffective and did not change clinical practice (3). One of the factors responsible for treatment resistance and impaired drug delivery is the challenging tumor microenvironment (TME) with extensive desmoplasia, a hallmark of PDA. The peritumoral stroma constitutes around 90% of the pancreatic tumor mass and is marked by an intense extracellular matrix deposition comprised of cancer-associated fibroblasts and a pro-tumoral local immunity (4, 5). However, strategies depleting fibroblasts in the TME resulted in deleterious patient outcomes highlighting the complex local regulation of stroma in PDA (6). Accordingly, the mere ablation of the pancreatic stroma itself seems to be clinically impractical.

So far, little is known about the immune microenvironment in the stromal compartment. A better understanding of immune regulation in the peritumoral stroma of PDA patients is needed to determine parameters of clinical significance. This could help to enhance treatment or stratification strategies and identify novel therapeutic targets.

In this study, we evaluated the tumor tissue of PDA patients after performing Laser Capture Microdissection allowing us to focus on the stromal compartment in particular. We systematically analyzed stromal tissues by serial immunohistochemistry as well as multiplex cytokine measurements and performed survival analyses. This led to the identification of IL9 and IL18 as critical stromal components with opposed clinical implications. Functional analyses shed light on the complex role of IL18 in the TME. Further, we assessed intercompartmental gradients of these cytokines in the tumor epithelium, stroma, and serum of our cohort. Finally, we compared serum levels of IL9 and IL18 in PDA patients with healthy individuals.

## Material and methods

### Patient cohort

The electronic pancreas database of the Department for General, Visceral and Transplantation Surgery at the University Hospital Heidelberg is prospectively maintained. It was searched for patients undergoing pancreatic surgery between 03/2007 and 07/2011. PDA patients without prior treatment, available frozen tumor tissue samples, presurgically obtained blood samples and available clinical baseline and outcome parameters were included. The local ethics committee of the University of Heidelberg approved sample and data collection. All patients signed written informed consent. Reference serum samples from healthy individuals (n=30) were provided by the blood bank of the University Hospital Heidelberg.

### Data collection

The following baseline data were extracted from the prospective database: Gender, age, weight and body mass index. The overall survival was also assessed. Pathological reports included pTNM tumor stage according to the TNM Staging Manual, American Joint Committee on Cancer (AJCC), tumor grading and resection margin status (7).

### Immunohistochemistry

The following mouse monoclonal antibodies were used to stain serial sections (4 µm) of cryopreserved PDA tissues and/or FFPE 3D bioprints: CD3ϵ (clone ab16669, 1:100, Abcam, UK; RRID: AB_443425), CD4 (clone 4B12, 1:150, Leica, Germany; RRID: AB_563559), CD8 (clone 4B11, 1:100, Leica, Germany; RRID: AB_442068), CD20 (clone L26, 1:100,Leica, Germany; RRID: AB_442055), CD163 (clone EPR19518, 1:500, Abcam, UK; RRID: AB_2753196), NKp46 (clone 195314, 1:175, R&D Systems, USA; RRID: AB_2149153), FoxP3 (clone 236A/E7, 1:100, Thermo Fisher Scientific, Germany; RRID: AB_467555), IL9 (clone EPR23484-151, 1:100, Abcam, UK), IL18 (clone EPR19954-188, 1:100, Abcam, UK), Granzyme B (clone 23H8L20, 1:200, Thermo Fisher Scientific, USA), Ki67 (clone MIB-1, 1:200, Dako, USA), LCK (clone 3A5, 1:50, Santa Cruz Biotechnology, USA). The complete staining procedure was carried out on a fully automated staining system (Bond-Max, Leica, Germany).

### Immunofluorescence

Double stainings were performed on FFPE patient tissues by using fluorochrome-conjugated secondary antibodies. The red fluorescence Alexa Fluor 594 donkey anti-mouse IgG (Thermo Fisher Scientific, Germany; A-21203) and the green fluorescence Alexa 488 goat anti-rabbit IgG (Thermo Fisher Scientific, Germany; A-11008) were used as indicated in the respected figures. First primary antibodies were IL9, IL18, CD3, CD68, CD4, CD8, CD163 and PD1. Stainings were performed according to antibody recommendations. The first primary antibody was incubated overnight at 4°C, then Alexa Fluor 488 (1:100 dilution) was applied for 60 minutes. The second primary antibody was applied for 180 minutes at room temperature and then detected with Alexa Fluor 594 (1:100 dilution) for 60 minutes. Cell nuclei mounting and staining was performed using Vectashield with DAPI (H-1200, 1:10000, Vector, USA). Images were scanned at 40-fold magnification with a Nanozoomer 2.0 HT scan system (Hamamatsu, Japan).

### Three-dimensional patient-derived tumor bioprinting

Pancreatic tumor cells were derived from patient ascites. Tumor cell containing ascites was placed in T25 cell culture flask, cultured at 37°C (5% CO_2_), and medium was replaced daily. Accutase (Merck, Germany) was used to detach outgrowing tumor cells, which were further expanded in a 1:1 mixture of cell-free ascites and DMEM (containing 10% FCS and 1% 94 penicillin/streptomycin). Also, PBMCs from a healthy donor were isolated from peripheral blood using Ficoll Paque Plus (GE17-1440-03, Merck, Germany) according to manufacturers’ instructions. The 3D bioprinting process was performed using the pneumatic extrusion-based bioprinter Bio X™ (Cellink, Goteborg, Sweden), as described before (8–10). Before printing, a cell suspension was created using a 1:1 mixture of PBMCs and patient-derived tumor cells in the respective culture medium. This cell suspension was then mixed 1:2 with the Cellink Bioink (Cellink, Sweden) to a final concentration of 2x10^6^ cells/ml. Multiple 3D droplets were printed in a 24-well plate according to manufacturer protocols (blue nozzle 22 gauge, pressure as required) and then crosslinked with Crosslinking Agent (Cellink, Sweden), containing 50 mM CaCl_2_, for five minutes. Afterwards, the bioprints were cultured in DMEM at 37°C (5% CO_2_) for 3, 5, 7 and 9 days. Media was changed regularly after 48 hours. Untreated bioprints were matched with bioprints treated with IL18-BP (3 µg/ml). Upon harvesting, the bioprints were cryopreserved and formalin-fixed.

### Cytokine measurements in serum, stromal tissue compartment and 3D bioprints

First, serial sections (20 µm) of cryopreserved PDA tissue samples were stained with cresyl-violet. Then, Laser Capture Microdissection was performed with the Leica Laser Microdissection V5.0.2.0 software to separate the stromal compartment from tumor epithelium. The procedure was performed according to the protocol of the inventors (11). Both, the stromal tissue as well as the 3D bioprints were lysated using the BioPlex™ Cell Lysis Kit (Bio-Plex Cell Lysis Kit, BioRad, USA; 171304011) according to manufacturer´s instructions. Serum samples were thawed overnight at 4°C and diluted 1:1 prior to protein quantification (Sample Diluent, BioRad, USA). All proteins of interest were concurrently quantified using a two-laser array reader. Concentrations were calculated with Bio-Plex Manager 4.1.1 based on a 5-parameter logistic plot regression formula. Bio-Plex Pro Human Cytokine Screening Panel 48-plex (BioRad, USA; 12007283), Bio-Plex Pro Human Cytokine ICAM-1 (BioRad, USA; 171B6009M) and Bio-Plex Pro Human Cytokine VCAM-1 (BioRad, USA; 171B6022M) were utilized to quantify cytokine concentrations.

### Quantification of immune cells

All tissue slides were scanned at 40-fold magnification. Whole-slide images were obtained with NanoZoomer 2.0 HT scan system (Hamamatsu, Japan). Cell quantification was performed with a software-based image analyzing system (VIS software suite, Visiopharm, Denmark). Estimation of cell density in cell conglomerates was carried out as described before (12–16). Quantification algorithms were used individually for each staining protocol according to the intensity of DAB staining.

### Statistical analysis

Statistical analyses were performed with GraphPad Prism 8.4.1. For the comparison of non-paired groups Mann Whitney tests were used. For paired sample analysis, Wilcoxon signed-rank tests were applied. Friedman test was used to detect differences in multiple samples. Non-normality distribution tests were applied, and non-parametric testing was used throughout. Overall survival time was determined by using the latest information. To perform survival analyses, patients were stratified into two groups (“high” and “low”) by determining the median cutpoints of cytokine concentrations or cell densities. Visualization of survival curves was carried out by using Kaplan Meier estimators. For survival analyses, patient groups were compared using the log-rank test. Based on the stromal cytokine concentration data we constructed correlation networks for IL9 and IL18 using Cytoscape software (17). The edge weights of the network are based on the Pearson correlation coefficient between cytokine-cytokine (r > 0.5, p < 0.05). All correlations with r > 0.5 are displayed. Differences were judged to be statistically significant in case of a p-value ≤0.05 and represented as follows: *=p ≤ 0.05, **=p ≤ 0.005 and ***=p ≤ 0.0001.

## Results

### Patient characteristics

51 PDA patients were included in our analysis. Blood samples were obtained before surgery and PDA samples after resection. As provided in **Table 1**, there were 26 men (51%) and 25 women (49%) in the cohort with a mean age of 65.6 (± 1.4). In all patients, histopathological evaluation confirmed the diagnosis of PDA. 50 patients (98%) had the tumor status pT3 and 1 patient (2%) had pT4. All patients were previously untreated.

**Table 1:** Patient characteristics.

Characteristic		Total (n=51)
**Male (n)**		26
**Female (n)**		25
**Age (years)**		65.6 ± 1.4
**Body weight (kg)**		71.45 ± 1.76
**BMI**		24.46 ± 0.57
**T**	T3 (n)	50
	T4 (n)	1
**N**	N0 (n)	8
	N1 (n)	43
**M**	M0 (n)	48
	M1 (n)	3
**Grade**	G2 (n)	33
	G3 (n)	18
**R^a^**	R0 (n)	6
	R1 (n)	44

Data are shown as mean ± SEM, BMI= body mass index (calculated as weight in kilograms divided by height in meters squared), T= stage of primary tumor, N= regional lymph node status, M= distant metastasis status, R= resection margin status. ^a^Information available on resection margin status from 50 patients.

### Stromal expression of IL9 and IL18 is associated with survival of patients

To systematically investigate the relevance of immunological properties in the peritumoral stroma in PDA, we first separated the stroma from tumor epithelium in PDA tissues of patients (n=51) using Laser Capture Microdissection. Then, a plethora of immune-related cytokines were analyzed in this compartment and survival analyses were conducted.

**Figure 1:**
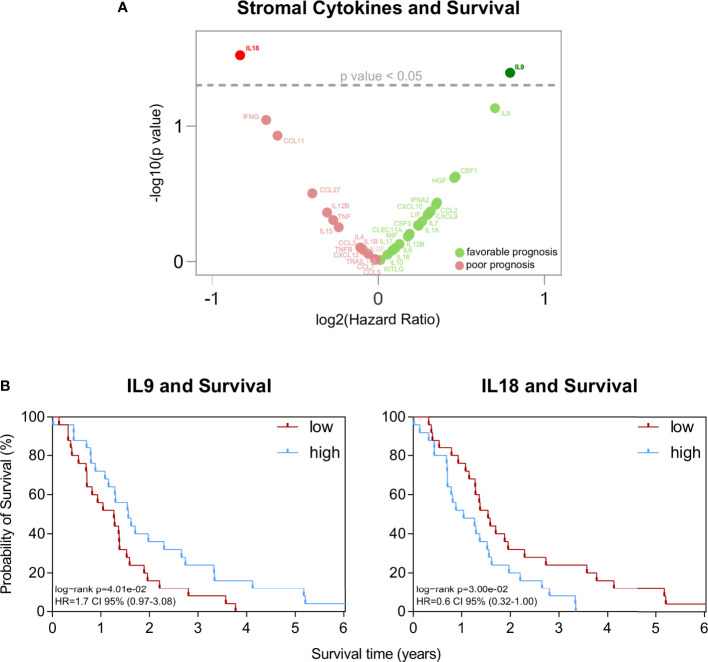
**(A)** Volcano plot of statistical significance (y-axis) against log2 (Hazard Ratio) (x-axis) for stromal cytokines. **(B)** Kaplan-Meier survival plots of patients with high versus low stromal concentration of IL9 (n=25/n=26) and IL18 (n=25/n=26). The median cutpoints were determined to stratify patients into high and low. Survival data were analysed using the log-rank test.

This assessment highlighted two stromal cytokines with significant association with survival in patients: IL9 and IL18 **(** **Figures 1A, B** **)**. While IL9 associated with improved survival (p=0.040), IL18 had the opposite effect (p=0.030) on patient outcomes **(** **Figure 1B** **)**. None of the other cytokines did relate to patient survival **(** **Figure 1A** **)**. Neither the sole tumoral expression nor the serum levels of IL9 and IL18 did associate with patient survival **(** **Figure S1A** **)**.

### The immune cell composition in the stroma differs from the tumor epithelium

To complement the cytokine analyses and assess the stromal immune cell presence, we systematically performed immunohistochemistry in the same PDA tissues using a variety of immune cell markers (CD3, CD4, CD8, FoxP3, CD20, CD163, NKp46) and quantified positively stained cells. Further, we compared their stromal density with the corresponding tumor epithelium.

**Figure 2:**
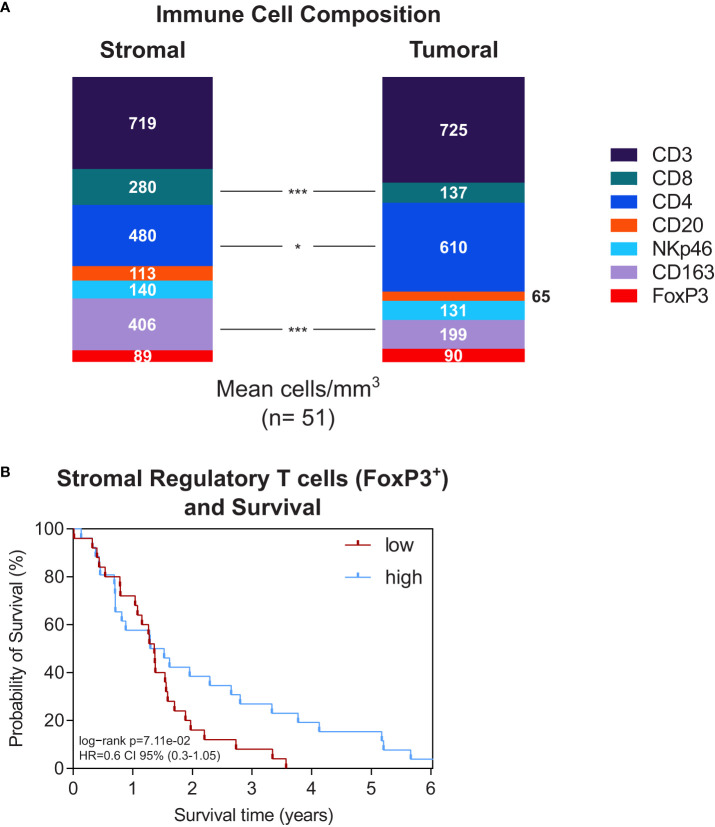
**(A)** Stacked bar plot of immune cell composition (as indicated) in the stroma (left) and tumor epithelium (right). Whole-tissue slides of n=51 PDA patients were analysed. The mean total immune cell density was 2227 cells/mm^3^ in the stroma and 1957 cells/mm^3^ in the tumor epithelium. *p≤0.05, ***p≤0.0001. **(B)** Kaplan-Meier survival plots of patients with high versus low stromal density of regulatory T cells (n=25/n=26) and IL18 (n=25/n=26). The median cutpoints were determined to stratify patients into high and low. Survival data were analysed using the log-rank test.

The total average number of positively stained immune cells was slightly higher in the stromal compartment (1957 cells/mm^2^ in the tumor epithelium *vs*. 2227 cells/mm^2^ in the stroma). However, focusing on the composition of immune cells revealed clear differences between the tumoral and stromal compartment **(** **Figures 2A** and **S2A** **)**. CD3^+^ T cells dominated in both areas (725 cells/mm^2^ in the tumor epithelium *vs*. 719 cells/mm^2^ in the stroma) but more CD4^+^ T cells were found intratumorally (p=0.043). Whereas more CD8^+^ T cells and CD163^+^ macrophages were localized in the peritumoral stroma (CD8^+^: p<0.0001, CD163^+^: p<0.0001) **(** **Figures 2A** and **S2A** **)**.

Next, we performed survival analyses to see whether stromal immune cell presence was related to patient outcome. The stromal density of the different immune cell types did not significantly associate to patient survival **(** **Figure S2B** **)**. However, an interesting aspect was the stromal presence of FoxP3^+^ regulatory T cells showing a tendency towards improved survival (p=0.071) with diverging curves in Kaplan Meier analysis **(** **Figure 2B** **)**.

### IL9 and IL18 correlate with distinct stromal immune phenotypes

To better understand the role of IL9 and IL18 in the stromal microenvironment of PDA, we compared groups with high *vs*. low stromal IL9 or IL18 concentration regarding other immune parameters (immune cell densities, correlation with other stromal cytokines).

**Figure 3:**
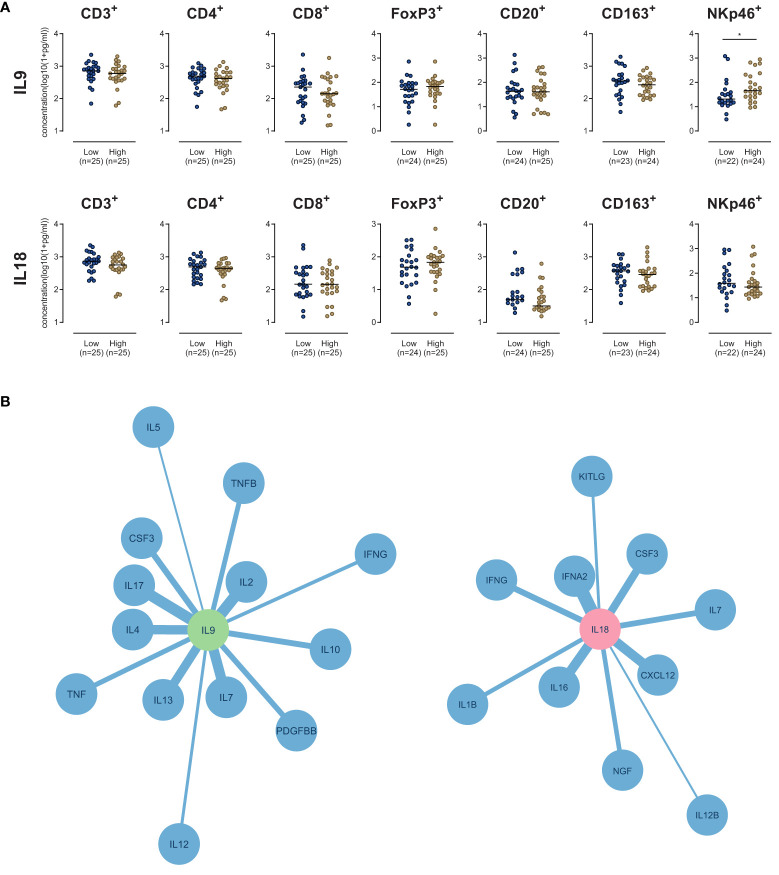
**(A)** Scatter dot plots comparing the density of immune cells (as indicated) in the stroma of PDA patients with high versus low IL9 or IL18. *p≤0.05. **(B)** Network plots of stromal cytokine-cytokine correlations of IL9 (Left) and IL18 (right) in human PDA tissue samples (n=51). All cytokines with a Pearson correlation coefficient r≥0.5 and p-value p<0.05 are shown. The edge weights of the network are based on the Pearson correlation coefficient.

Concerning IL9, only the density of NK cells showed a significant difference between both groups: High stromal IL9 levels were associated with higher NK cell density (p=0.037) **(** **Figure 3A** **)**. None of other analyzed immune cell types did show a difference in their density **(** **Figure 3A** **)**. Regarding IL18, no immune cell type was associated with its expression **(** **Figure 3A** **)**.

Stromal cytokine-cytokine correlation analyses showed that IL9 strongly correlated (r>0.5, p<0.05) with a subset of molecules: IL2, IL7, IL4, IL13, IL17, CSF3, IL10, TNF, PDGFBB, IFNG, IL12 and IL5 **(** **Figure 3B** **).** IL18 strongly correlated (r>0.5, p<0.05) with IFNA2, CXCL12, IL16, CSF3, IFNG, IL7, NGF, IL1B, KITLG and IL12B **(** **Figure 3B** **)**.

### Sources of IL9 and IL18 in the microenvironment

**Figure 4:**
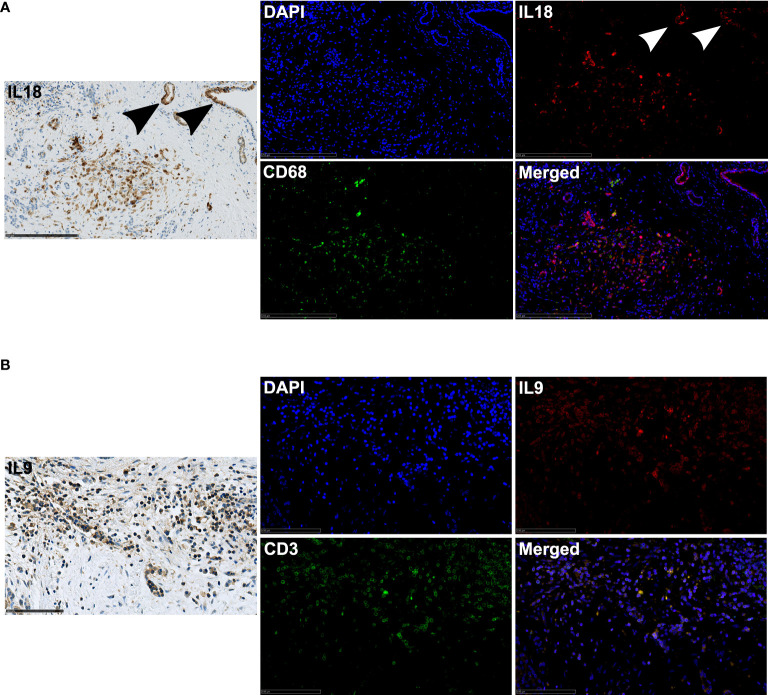
**(A)** Exemplary immunohistochemistry (IL18) and immunofluorescence (IL18/CD68) image staining in a PDA patient tissue. 20x magnification. Scale bar: 250µm. Arrows highlight positively stained activated pancreatic ducts. **(B)** Exemplary immunohistochemistry (IL9) and immunofluorescence (IL9/CD3) image staining in a PDA patient tissue. 40x magnification. Scale bar: 100µm.

To identify the spatial sources of IL9 and IL18 in the microenvironment, we performed multiple immunohistochemical and immunofluorescence stainings in tissues of PDA patients. We demonstrated that IL18 is secreted by different cell types in the microenvironment by staining serial sections of the same tissue: activated pancreatic duct cells, CD68^+^ macrophages **(** **Figure 4A** **)** and also CD163^+^ macrophages **(** **Figure S4A** **)**. In contrast IL9 is primarily expressed by CD3^+^ T cells, accordingly CD3^+^/IL9^+^ T cells represent Th9 cells **(** **Figure 4B** **)**.

### IL18 associates with T cell exhaustion and its inhibition in patient-derived tumor bioprints diminishes Th1- and Th2-type cytokines

**Figure 5:**
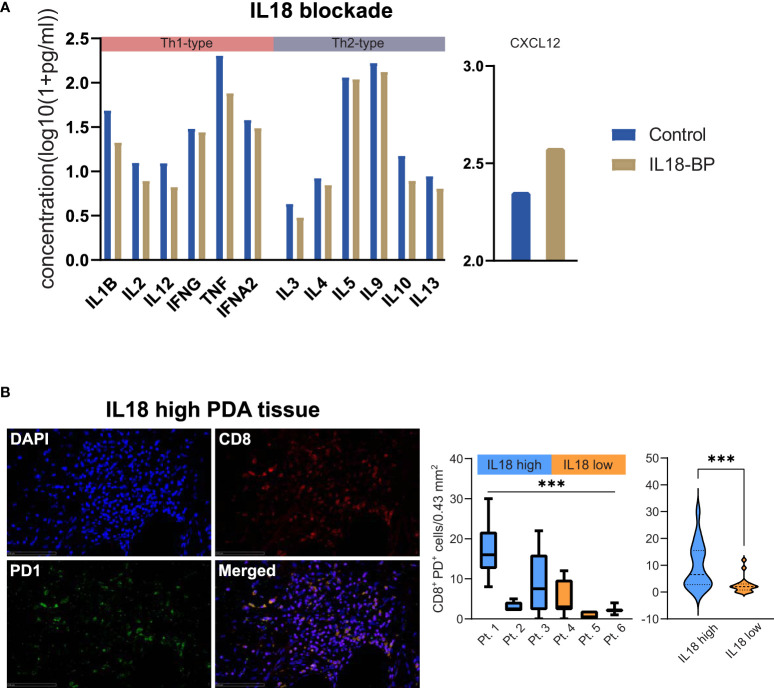
**(A)** Bar plots showing levels of cytokines (as indicated) in a patient-derived PDA bioprint treated with IL18-BP (IL18 blockade) vs. untreated control. Cytokines were categorized in TH1- and TH2-type cytokines (as indicated). **(B)** Exemplary immunofluorescence image of CD8/PD1 staining in a PDA patient tissue with high stromal IL18 levels. 40x magnification. Scale bar: 100µm. Box plots comparing the density of CD8^+^PD1^+^ T cells in individual PDA patient tissues with high vs. low levels of IL18 are displayed. Further, violin plots comparing the density of CD8^+^PD1^+^ T cells in high vs. low IL18 patient groups are shown. ***p≤0.005. PDA=Pancreatic ductal adenocarcinoma.

To assess the functional impact of IL18 in PDA, we blocked IL18 using IL18-binding protein (IL18BP) in a patient-derived model using tumor bioprints. The bioprints consisted of patient-derived pancreatic tumor cells as well as PBMCs embedded in a bioink which consists of non-animal derived polysaccharide components. Blocking IL18 resulted in downregulation of Th1-type (IL1B, IL2, IL12, IFNG, TNF, IFNA2) as well as Th2-type (IL3, IL4, IL5, IL9, IL10, IL13) cytokines **(** **Figure 5A** **)**. On the opposite, CXCL12 levels increased upon IL18 blockade **(** **Figure 5A** **)**. Staining analyses of cytotoxic markers (LCK, Granzyme B) or the proliferation marker Ki67 did not show different expression patterns upon IL18 inhibition **(** **Figure S5A** **)**. To investigate whether IL18 affects T cell plasticity in the pancreatic TME, we assessed the T cell exhaustion status in patient tissues. The analysis demonstrated that tissues with high stromal IL18 levels have a clearly higher expression rate of CD8^+^/PD1^+^ T cells than tissues with low IL18 expression **(** **Figure 5B** **)**.

### Assessment of intercompartmental gradients of IL9 and IL18

The functionality of cytokines in complex environments depends on gradients between different areas. To consider the role of spatial concentrations and gradients of IL9 and IL18, we investigated the tumor-to-stroma and stroma-to-serum gradients of IL9 and IL18 in our patient cohort. We compared the gradients in groups with high *vs*. low stromal concentration of IL9 or IL18.

**Figure 6:**
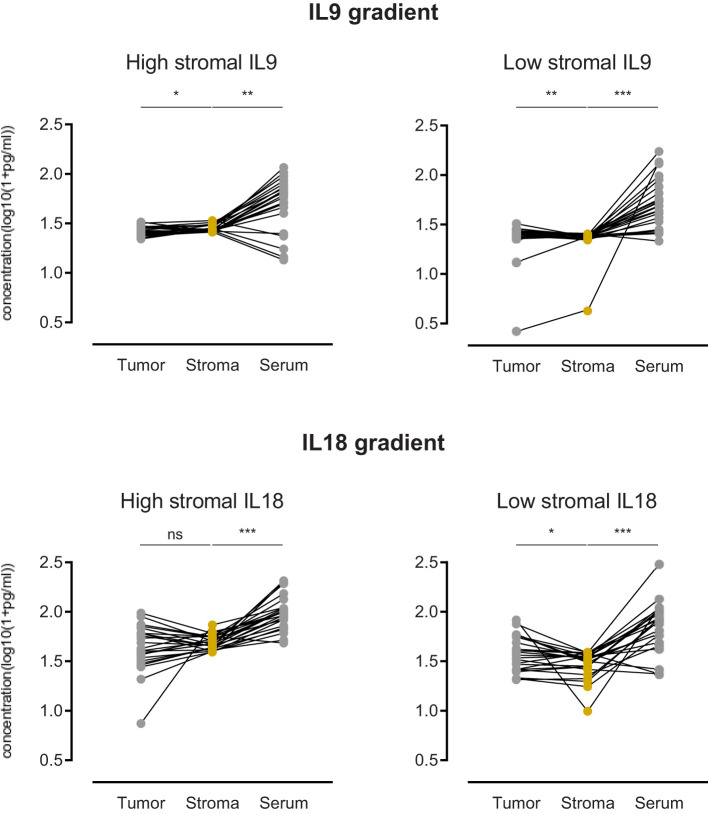
Comparison of PDA patients with high stromal IL9 or IL18 (left) (n=25) versus low stromal IL9 or IL18 (right) (n=26) regarding the gradient of cytokine concentration in three compartments: tumor epithelium, stroma, serum. ns=not significant, *p≤0.05, **p≤0.005, ***p≤0.0001.

In the case of IL9, we revealed that patients with high stromal IL9 levels (prognostic beneficial) show a significant tumor-to-stroma IL9 gradient towards the stroma **(** **Figure 6** **)**. In contrast, patients with low stromal IL9 levels showed a tumor-to-stroma IL9 gradient towards the tumor epithelium **(** **Figure 6** **)**. In both groups (low and high stromal IL9) the stroma-to-serum gradient was significantly directed towards the serum **(** **Figure 6** **)**.

Focusing on IL18, we found that patients with low stromal IL18 levels (prognostic beneficial) show a significant tumor-to-stroma gradient away from the stroma (and towards the tumor epithelium) **(** **Figure 6** **)**. While patients with high stromal IL18 levels did not display a relevant gradient **(** **Figure 6** **)**. As seen for IL9, both groups (low and high stromal IL18) exhibited a stroma-to-serum gradient significantly directed towards the serum.

### Comparison of circulatory IL9 and IL18 levels in PDA patients and healthy individuals

To determine whether circulatory levels of IL9 or IL18 in PDA patients differ from healthy individuals and whether they potentially reflect systemic tumor responses, we compared IL9 and IL18 blood levels of our patient cohort with an age-matched control group of healthy individuals (n=30).

**Figure 7:**
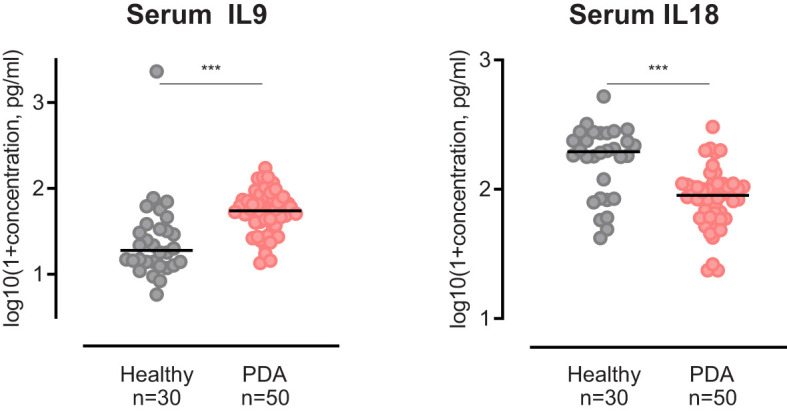
Scatter dot plots comparing Serum IL9 and Serum IL18 concentration of PDA patients versus healthy controls. ***p≤0.0001.

Our analysis highlighted that serum IL9 levels in PDA patients were significantly elevated in comparison to healthy subjects **(** **Figure 7** **)**. In contrast, serum IL18 levels in PDA patients were significantly lower than in the control group **(** **Figure 7** **)**.

## Discussion

The development, progression and treatment resistance in pancreatic ductal adenocarcinoma (PDA) are dependent on the composition and functionality of the circumjacent microenvironment. The surrounding immune cells, fibroblasts, other non-tumor cells, and extracellular matrix make up most of the tumor volume in PDA (5). But the role of the peritumoral stroma remains controversial: on one hand it forms a barrier which prevents malignant cells from spreading, but on the other hand the dense stroma impedes sufficient drug delivery to the tumor site (18). Thus, it is worthwhile to investigate the role of the stroma in PDA: promoter or inhibitor of disease. Accordingly, targeting of the stroma as a therapeutic approach can yield unexpected consequences. Of further note, the cellular and acellular stromal immunity plays a pivotal role in controlling pancreatic tumor biology (19). Cellular components like immune or tumor cells mainly execute functions by secretion of cytokines determining the phenotype of the tumor microenvironment (TME) and thereby influence patient outcomes. Deciphering the immune features of stroma in PDA patients is important to better understand the complex tumor biology, identify potential exploitable targets and improve clinical management strategies overcoming the present gap between preclinical data and actual patient care.

Our systematic analysis has revealed for the first time that stromal expression of IL9 and IL18 in PDA patients associate with patient outcomes. However, they have opposed prognostic significance: while stromal IL9 is beneficial, IL18 has a detrimental implication. IL9 is a pleiotropic molecule, known as a multifaceted regulator of immune responses in different cell types. In the past, preclinical results from cell experiments described IL9 as a tumor-supportive cytokine promoting proliferation and metastasis in pancreatic cancer cells (20). And a targeted blockade of IL9 led to a restrained tumor growth in a murine model of pancreatic cancer (21). However, the role of IL9 has been revisited since the discovery of T helper (Th) 9 cells. It has been shown that Th9 cells produce high amounts of IL9 and unfold multiple IL9-dependent anti-tumoral effects in solid cancers (22–25). We confirm that Th9 cells in the pancreatic TME are the primary source of IL9. And our results confirm the potential beneficial properties of IL9 arising from the peritumoral stroma in PDA patients. Further, we demonstrate that IL9 expression associates with stromal NK cell density. This fits into previous reports, where IL9-producing Th9 cells induced cytolytic and secretion functions in NK cells, a mechanism orchestrated *via* IFNG (26). But densities of other immune cells were not related to stromal IL9 or IL18 levels suggesting that these cytokines fulfill immune regulatory functions rather than affecting numbers of immune cells in the pancreatic TME. Another possibility might be that primarily non-immune cells are targeted. We demonstrate that stromal IL9 is embedded in a network of known Th9 cell regulatory cytokines (IL2, IL4, IL7, IL10) as well as inflammatory and Th1-type molecules (IL12, IFNG, TNF, TNFB) (26) underscoring the interconnection of IL9 with a predominantly anti-tumoral local immune phenotype of the stroma. Another observation is the strong correlation of stromal IL9 with IL17 expression indicating a link to Th17 cell regulation in PDA. Previous work has shown this anti-tumoral interconnection in preclinical model systems (27).

Our work highlights stromal IL18 as a marker for poor prognosis in PDA patients. So far, the role of IL18 in PDA is unclear, even more so, when focusing on the peritumoral stroma. Based on the available evidence, diverse and opposing effects of IL18 have been suggested. As described before (28, 29), we demonstrate by staining serial sections that IL18 was mainly expressed by M1-like (CD68^+^/CD163^-^) tumor-associated macrophages (TAM) but also M2-like (CD68^+^/CD163^+^) TAMs in our cohort. From a functional point of view, on the one hand, IL18 is supposed to promote progression and invasiveness of pancreatic cancer cells. Immunohistochemical approaches show that tumoral IL18 relates to poorer outcomes (30, 31). Also, high blood IL18 levels correlate with worse patient survival (32). On the other hand, IL18 potentially acts as a stimulator of Th1-type responses (*via* IL12), increases IFNG concentrations and thereby exhibits anti-tumoral features (33, 34). But recombinant IL18 did not lead to therapeutic effects in clinical trials (35). One possible reason might be the high-affinity IL18 decoy receptor, which limits anti-tumor activity in preclinical studies (36). In our cohort, while IL18 correlates with a subset of Th1-type cytokines (IFNG, IFNA2, IL12B), it is also strongly linked to CXCL12 expression. The link of IL18-promoted secretion of CXCL12 in fibroblasts is known from autoinflammatory diseases (37). And the CXCL12/CXCR4 axis in PDA is a promoter of tumor proliferation, invasion and chemoresistance (38–41). Furthermore, clinical approaches indicate that CXCL12 inhibition promotes a beneficial Th1 type tissue reactivity in PDA patients (42). Our functional analyses show that inhibiting IL18 diminishes both Th1- and Th2-type cytokines, while CXCL12 expression increases upon IL18 blockade. The effect on T cell phenotypes is not surprising as IL18 is known to facilitate both Th1- and Th2-type responses (43). Staining analyses showed no upregulation of classical activation markers of T cells (LCK, Granzyme B). This underlines the complex functionality of IL18 in the pancreatic TME and our data provides evidence that stromal IL18 weakens anti-tumoral immune responses in PDA. This is further underscored by the association of IL18 with T cell exhaustion in our study. In light of this, it seems that the balance of Th1 and Th2 through the regulation *via* IL18 is more leaning towards Th2 with more T cell exhaustion markers present. Ultimately, our data underlines that using IL18 as therapeutic approach must be carefully considered in PDA patients as stromal IL18 associates to worse patient outcomes.

The description of specific compartmental effects of IL9 and IL18 in the stroma cannot be concluded without looking into the tumor compartment in comparison. In this direction, our analysis of the tumor compartment alone shows no prognostic significance for IL9 or IL18 concentrations. Moreover, also serum levels of IL19 or IL18 are not associated with patient survival. As the complex interplay and regulation of immunological processes can also be limited to the stroma, separate investigations show an interdependency of the overall prognostic effect in relation to the linked tumor-to-stroma cytokine gradients. It has been demonstrated in the past that the mere focus on cytokine concentrations in one compartment does not reflect the full picture. Knowledge of the compartmentalization and corresponding cytokine gradients is needed to comprehensively recapitulate tumor biology (44). This is especially true for the “intra-patient” view on the concentration differences between the stroma and the tumor. In light of the overarching “high versus low” classification, our intra-patient view on the direction of the gradient provides further insight and allows better stratification. In case of IL9, patients with high stromal expression (favorable prognosis) have their tumor-to-stroma IL9 gradient directed towards the stroma. And the opposite observation is made in patients with low stromal IL9 expression. This underlines that not only a high stromal IL9 expression is beneficial, but the concentration must apparently be higher than in the tumor epithelium. Conversely, this conclusion can also be drawn for IL18. Patients with low stromal IL18 expression (favorable prognosis) have their tumor-to-stroma gradient directed away from the stroma towards the tumor epithelium. These findings underline that compartmental cytokine measurements allow a more comprehensive assessment of the stroma biology and immune microenvironment.

Accordingly, our results show that the immune cell composition also varies clearly in both compartments. Cytotoxic T lymphocytes and tumor-associated macrophages prevail in the stroma while CD4^+^ T cells are predominantly found in the tumor epithelium. Focusing on the prognostic potential of stromal immune cells, no cell type significantly associates to patient survival. However, a tendency of stromal FoxP3^+^ regulatory T cells towards beneficial outcomes is noted. This seems counterintuitive, but our observation fits to recent data where depletion of regulatory T cells related to fibroblast-associated acceleration of pancreatic carcinogenesis (45). Also, significant levels of regulatory T cell densities are associated with better survival in colorectal cancer (46). In light of a sole stromal distribution, the prognostic effect of increased presence of T cells (e. g. CD8^+^ T cells) is limited and appears to be more relevant in the tumor compartment with direct contact to tumor cells (47–49).

Interestingly, serum analysis in our patients again reveal an opposing finding for IL9 and IL18. Circulatory levels of IL9 in PDA patients are higher than in healthy individuals, in contrast, IL18 levels are lower than in the control group. A possible explanation for this observation is the tumor-induced recruitment of circulatory IL18 and its accumulation in the pancreatic stroma which contributes to tumor progression and poor patient outcomes. However, the functionality of these cytokines, their diagnostic potential as well as prognostic significance need to be further investigated in prospective studies.

IL9 is knowingly regulated by microbial communities and host-microbiota interactions are necessary to induce antitumoral IL9 functions (50–52). Also, mounting evidence suggests that IL18 is regulated by local microbial composition and changes induced by antibiotic treatment or other treatments (like chemotherapy) and therefore can be regarded as an influential dynamic factor in tumor biology (53). Further studies are needed to better understand the role of the pancreatic local microbiome in regulation of stromal IL9 and IL18 functionality.

In summary, our work provides new insights into the complex regulation of stromal immunity in PDA patients. Using human PDA tissue allowed us to recapitulate the situation in patients considering all components of the microenvironment. The identification of IL9 and IL18 as key stromal cytokines with prognostic significance shed light on clinically exploitable parameters. For instance, stromal IL9 could serve as patient stratification marker for immunotherapeutic strategies or as a possible treatment agent in combination with stroma-targeting therapies. The current data help to clear the -sometimes conflicting- findings for these two cytokines in their role within the microenvironment. Differential investigation on sub-compartments helps to shed light on dichotomous effects of cytokines. As this has relevance for clinical approaches, it is advisable to integrate these aspects before applying e. g. IL18-based therapies in PDA patients.

## Data availability statement

The original contributions presented in the study are included in the article/**Supplementary Material**. Further inquiries can be directed to the corresponding author.

## Ethics statement

This study was reviewed and approved by Ethics Committee of the University of Heidelberg. The patients/participants provided their written informed consent to participate in this study.

## Author contributions

Conceptualization, AA and NH. Methodology, AA, SK and NH. Formal Analysis, AA and NH. Investigation, AA, SK and RK. Resources, RK, TH, NG, CS, DJ and NH. Data Curation, AA and NH. Writing - Original Draft, AA. Writing - Review and Editing, AA, RK, NH. Visualization, AA and NH. Supervision, NH. Project Administration, AA and NH. Funding Acquisition, DJ and NH. All authors contributed to the article and approved the submitted version.

## Funding

This work was supported by the RR Pohl Stiftung.

## Acknowledgments

All PDA and serum samples were provided by the Pancobank platform at the European Pancreas Center Heidelberg (EPZ), member of the Biomaterial Bank Heidelberg (BMBH). We thank Jana Wolf, Ulrike Prüfer and Rosa Eurich for their much-appreciated technical assistance.

## Conflict of interest

The authors declare that the research was conducted in the absence of any commercial or financial relationships that could be construed as a potential conflict of interest.

## Publisher’s note

All claims expressed in this article are solely those of the authors and do not necessarily represent those of their affiliated organizations, or those of the publisher, the editors and the reviewers. Any product that may be evaluated in this article, or claim that may be made by its manufacturer, is not guaranteed or endorsed by the publisher.
