# Supplementary figures and images for: Immune features of the peritumoral stroma in pancreatic ductal adenocarcinoma
Source: Front Immunol. 2022 Sep 5;13:947407. doi: 10.3389/fimmu.2022.947407 (PMC9483939; doi:10.3389/fimmu.2022.947407)

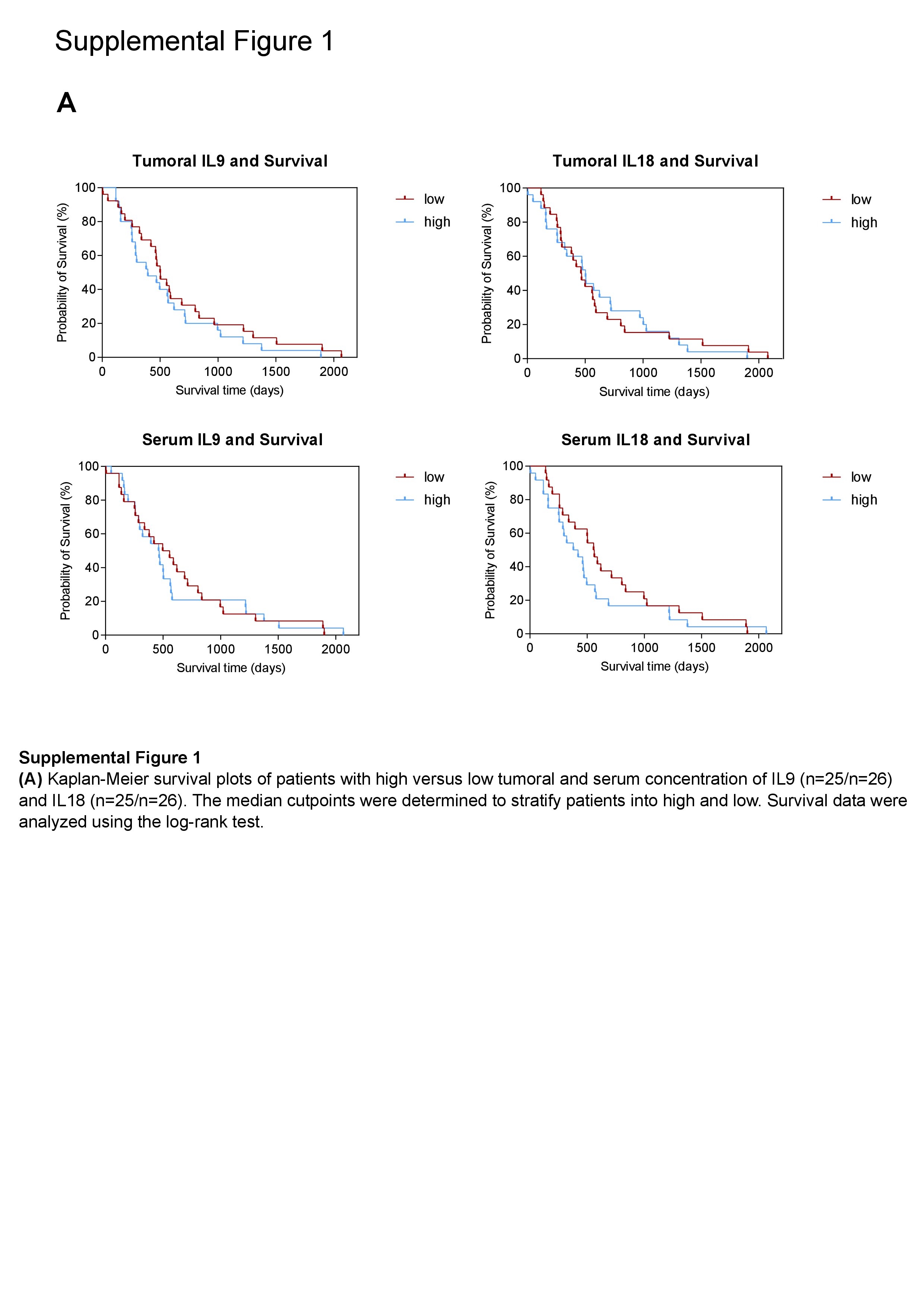

Supplement: Supplementary file 1 [file Image_1.jpeg]

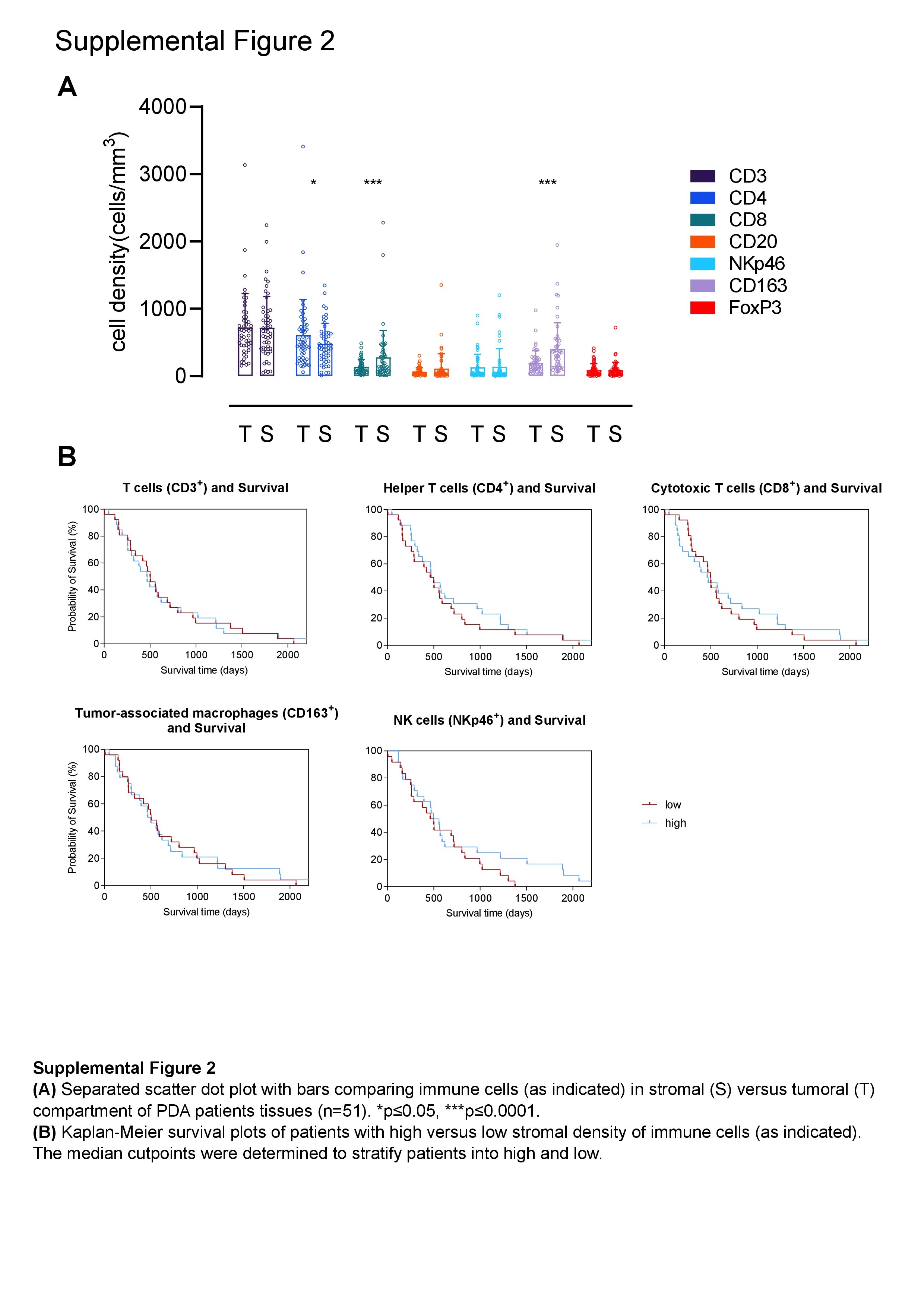

Supplement: Supplementary file 2 [file Image_2.jpeg]

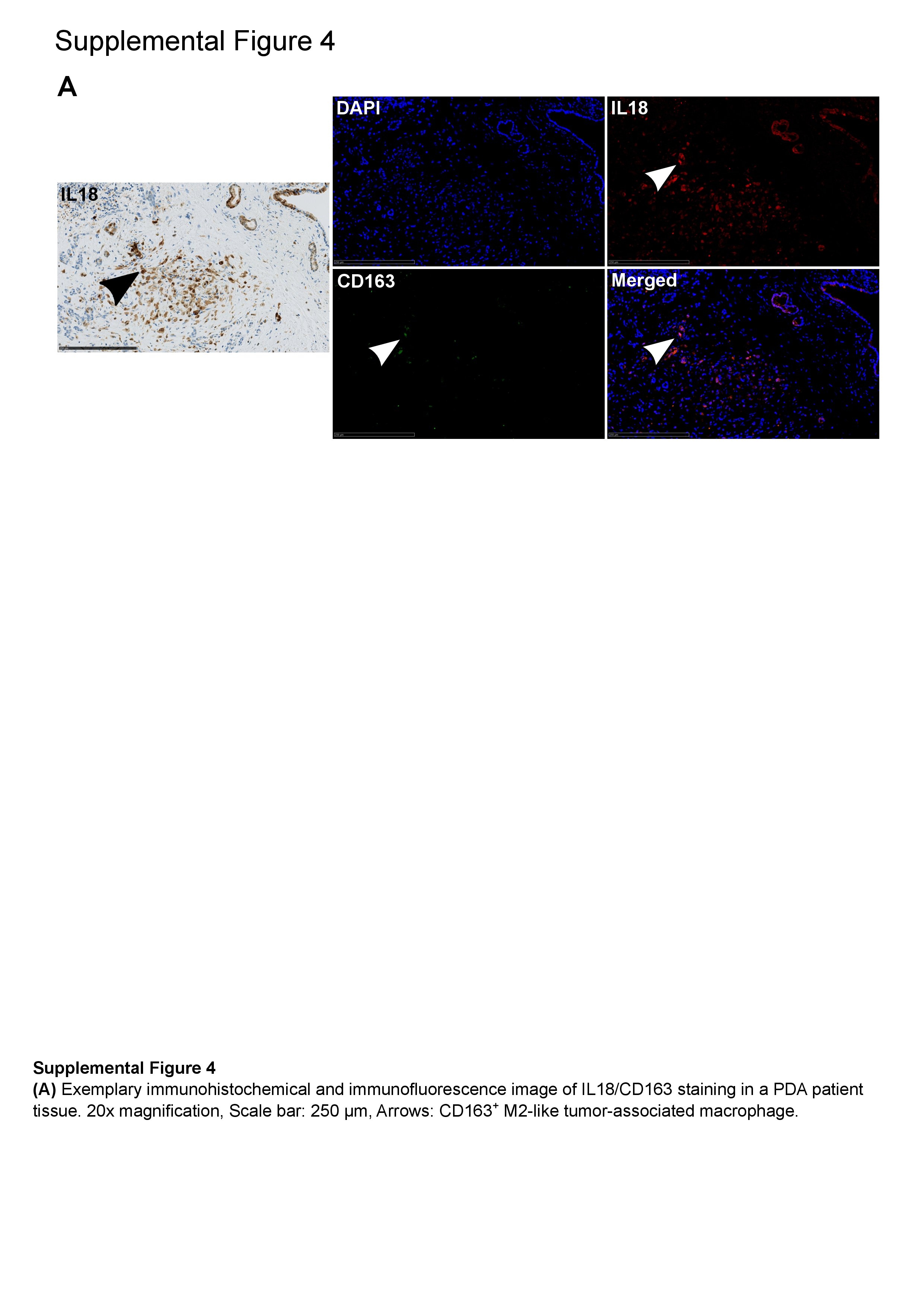

Supplement: Supplementary file 3 [file Image_3.jpeg]
